# Integrating heat stress tolerance indices based on grain yield for the selection of resilient wheat genotypes

**DOI:** 10.3389/fpls.2026.1949670

**Published:** 2026-10-27

**Authors:** Narain Dhar, Manish Kumar Vishwakarma, Uttam Kumar, Palaparthi Dharmateja, Neeraj Budhlakoti, Sundeep Kumar, Pradeep Kumar Bhati, Pradeep Kumar Sharma, Boddupalli Maruthi Prasanna

**Affiliations:** 1 Borlaug Institute for South Asia, New Delhi, India; 2 Department of Genetics and Plant Breeding, Chaudhary Charan Singh University, Meerut, India; 3 Astralyan Agro (OPC) Pvt. Ltd, Shamli, Uttar Pradesh, India; 4 ICAR-Indian Institute of Groundnut Research, Junagadh, India; 5 ICAR-Indian Agricultural Statistics Research Institute, New Delhi, India; 6 ICAR-National Bureau of Plant Genetic Resources, New Delhi, India; 7 CIMMYT-BISA, New Delhi, India

**Keywords:** cluster analysis, heat stress, MGIDI, multivariate analysis, tolerance indices, wheat

## Abstract

Wheat productivity is threatened by terminal heat stress, which hampers yields 6-10% per 1 °C above optimal temperatures during grain filling. Although heat stress tolerance indices offer objective selection criteria, their individual effectiveness varies with genetic background and environment. This study evaluated 230 diverse bread wheat genotypes across six environments (three locations × two seasons, 2021-23) under timely- and late-sown conditions to identify heat-tolerant germplasm using an integrated multivariate framework. Significant genotypic variation (*P*< 0.001) was detected for grain yield under non-stress (Yp), heat-stress (Ys) conditions, TOL, and STI, whereas HSI showed no significant genotypic effect. Correlation analysis revealed strong positive associations among the yield-derived stress tolerance indices, reflecting their shared dependence on grain yield under contrast conditions. PCA captured 95.23% of the total variation in the first two components, with Yp, Ys, STI, and TOL defining the major productivity-related dimension and HSI contributing predominantly to a susceptibility-related dimension. Hierarchical clustering separated the genotypes into three contrasting stress-response profiles. MGIDI selected 34 genotypes at 15% selection intensity, with 28 (82.4%) belonging to the cluster showing a comparatively favorable stress-response profile. Genotypes G10037, G10018, and G12015 showed consistent identification across index-based ranking, clustering, and multi-trait selection. These genotypes represent promising candidates for further evaluation under terminal heat-stress environments. The results demonstrate that combining complementary multivariate approaches can improve prioritization of wheat germplasm while avoiding overreliance on highly correlated yield-derived stress indices.

## Introduction

Wheat (*Triticum aestivum* L.) is among the most important cereal crops globally, providing approximately 20% of the total dietary energy and protein intake for billions of people (FAO, 2022). Cultivated across approximately 220 million hectares spanning temperate to subtropical agro-climatic regions, wheat is indispensable to global food security, particularly in developing countries. With the world population projected to reach 9.7 billion by 2050, demand for wheat continues to intensify, placing enormous pressure on production systems to achieve higher and more stable yields. Despite remarkable yield gains achieved through Green Revolution technologies viz., improved varieties, fertilizers input, irrigation, and integrated pest management. Notwithstanding, productivity growth has begun to plateau in many major producing regions in the world. Increasingly frequent and severe abiotic stresses are now recognized as primary constraints limiting further progress. Among these, heat stress has emerged as one of the most threatening, with its impacts projected to intensify under change in the climatic conditions.

Wheat is particularly sensitive to elevated temperatures during critical reproductive stages, including flowering, pollination, and grain filling. Optimal temperatures during the reproductive phase range from 15 °C to 22 °C. The temperatures exceeding 30 °C during anthesis and grain filling are generally considered stressful (Farooq et al., 2011). Terminal heat stress is the occurrence of high temperatures towards the end of the crop cycle during grain filling (Suryavanshi and Buttar, 2016). It accelerates senescence, reduces photosynthetic duration, disrupts carbohydrate metabolism, and impairs starch-synthesizing enzymes. The result is underdeveloped, shriveled grains of poor quality, directly undermining food security and farmer livelihoods. Asseng et al. (2015) estimated that global wheat yields decline by 6-10% for every 1 °C rise in temperature during the growing season.

For the identification of stress tolerant cultivars, several studies suggested different stress tolerance indices that can be computed from grain yield data in contrast (stress and non-stress) environments. Notwithstanding, some of them are useful for selection of heat tolerant genotypes in wheat and breeders can rely on heat stress tolerance indices. These indices include the Stress Tolerance Index (STI) (Fernandez, 1992), Heat Susceptibility Index (HSI) (Fischer and Maurer, 1978), Tolerance Index (TOL) (Rosielle and Hamblin, 1981). These indices integrate performance across environments and provide quantitative criteria for screening diverse germplasm. Several studies have demonstrated the usefulness of yield-based stress tolerance indices for differentiating wheat genotypes under terminal heat stress and contrasting sowing environments (Kumar et al., 2021; Poudel et al., 2021).

Despite these advances, the relative effectiveness of individual indices varies with genetic background, stress intensity, and environment, and no single index has proven universally superior for all germplasm and testing conditions. There is necessity for comprehensive, multi-environment evaluation of tolerance indices alongside advanced multivariate tools that can simultaneously account for multiple traits and environments. The Multi trait genotype ideotype distance index (MGIDI) by (Olivoto and Nardino, 2021) offers such a framework, ranking genotypes based on their proximity to a defined Ideotype across all measured traits, thereby reducing the bias inherent in single-index selection.

Considering above background, the present study has been designed to: (i) evaluate 230 diverse bread wheat genotypes under normal (timely sown) and heat stress (late sown) conditions across three locations over two years (2021–22 and 2022-23) (ii) compute and compare different heat stress tolerance indices viz. TOL, STI, and HSI (iii) examine associations among these indices and grain yield using correlation and principal component analysis (PCA); (iv) classify genotypes into tolerance groups through cluster analysis; and (v) identify the most heat-resilient genotypes using the MGIDI approach. This integrated framework aims to provide a robust and replicable methodology to identify terminal heat stress tolerant wheat genotypes.

## Materials and methods

### Field experiments

Field experiments were conducted over two consecutive rabi seasons (2021–22 and 2022-23) at the research stations of the Borlaug Institute for South Asia (BISA) in the three main wheat-growing regions of India: (1) Ludhiana, Punjab, representing the North-Western Plain Zone (NWPZ), (2) Jabalpur, Madhya Pradesh representing the Central Zone (CZ), and (3) Pusa, Samastipur, Bihar representing the North-Eastern Plain Zone (NEPZ). The geographic coordinates, altitude, and soil types for each site are mentioned in **Table 1**.

**Table 1:** Site details, production environments, and codes used during 2021–22 and 2022-23.

Location	Code	Sowing condition	Irrigation & fertilizer	Season/year	Coordinates & altitude, soil
BISA Jabalpur	E1	Normal & Stress Environment	Sprinkler (5) NPK (RFD-120:60:40)	2021-22	23°10’N, 79°59’E; 394 masl Black cotton-clay Soil
	E2	Normal & Stress Environment	Sprinkler (5) NPK (RFD-120:60:40)	2022-23	
BISA Ludhiana	E3	Normal & Stress Environment	Flood -NPK (RFD-150:60:40)	2021-22	(30°59′N, 75°45′E; ~247 masl), Sandy Loam to Loam Soil
	E4	Normal & Stress Environment	Flood -NPK (RFD-150:60:40)	2022-23	
BISA Samastipur	E5	Normal & Stress Environment	Flood-NPK (RFD-120:60:40)	2021-22	25°57′N, 85°40′E; 52 masl Calcareous Alluvial Soil
	E6	Normal & Stress Environment	Flood-NPK (RFD-120:60:40)	2022-23	

masl, meter above sea level.

At each location, crops were planted on two sowing dates to create contrast thermal environments. In the timely-sown (non-stress) treatment, crops were planted during the second week of November, ensuring that the crop reached its reproductive and grain-filling stages under near-optimal temperatures. In the late-sown (heat-stress) treatment, sowing was delayed by approximately four weeks to the third week of December, so that flowering, grain filling, and maturity impose to more than optimal temperatures during month of March and April. The late-sown treatment is a well-established approach to impose terminal heat stress under field conditions. Temperature data (mean daily maximum and minimum) recorded at each location during both seasons are summarized in **Supplementary Figure 1**. Flowering and maturity dates defined the grain-filling period, during that period temperature were recorded. Mean Tmax, maximum Tmax, mean Tmin, and frequencies of Tmax >30 and >35 °C were recorded. For daily records, cumulative heat exposure was calculated as $\sum \text{max}\;(T_{\text{max}},-30,0)$ (°C-days); for LDH 2022-23, weekly observations were retained.

### Plant materials

A panel of 230 diverse bread wheat genotypes were used for the study, comprising 228 accessions obtained from the ICAR-National Bureau of Plant Genetic Resources (NBPGR), New Delhi, and two standard check cultivars, RAJ3765, and HD2932. The checks were included to provide reference control benchmarks under both sowing conditions. Detail information of genotypes along with pedigree information has been provided in **Supplementary Table S1**.

### Experimental design

The experiment was laid out in two replications in a Lattice design. Each genotype was sown in a plot of 3.024 m² comprising six rows of 2.8m length with row-to-row distance 18cm. All standard management practices including fertilizer doses, irrigation, and plant protection measures were followed for each location as provided in **Table 1**.

### Computation of heat tolerance indices

To assess the performance of wheat genotypes under heat-stress and non-stress environments, a set of widely used heat-tolerance indices was computed from grain yield data. These indices allow identification of genotypes that maintain high and stable yields under terminal heat stress. The indices, their formulae, and original references are as follows. In all formulae, Yp = grain yield of a genotype in the non-stress (timely-sown) environment; Ys = grain yield of the same genotype in the heat-stress (late-sown) environment; $\bar{y}$p = mean grain yield of all genotypes in the non-stress environment; $\bar{y}$s = mean grain yield of all genotypes in the heat-stress environment.

Tolerance Index (TOL) TOL = Yp − Ys. Measures absolute yield reduction under stress; lower values indicate greater tolerance (Rosielle and Hamblin, 1981).

Stress Tolerance Index (STI) = (Yp × Ys)/( $\bar{y}$p)². Identifies genotypes with superior performance in both conditions; higher values are preferable (Fernandez, 1992).

Heat Susceptibility Index (HSI) = [1 - (Ys/Yp)]/D, where D = 1-( $\bar{y}$s/ $\bar{y}$p). Evaluates susceptibility to heat stress; lower values indicate greater tolerance (Fischer and Maurer, 1978).

Combined analysis of variance was performed across the six environments using genotype and environment as fixed effects: $Y_{ij}=\mu +G_{i}+E_{j}+\epsilon_{ij}$. Because one genotype-level observation was available for each genotype × environment combination, genotype × environment interaction was not estimated separately and was included in the residual term. Pearson correlation, principal component analysis, hierarchical clustering, MGIDI, and joint ranking were subsequently used to characterize relationships and prioritize genotypes based on Yp, Ys, TOL, STI, and HSI. All stress indices were computed in Microsoft Excel, Analysis of variance (ANOVA), Pearson’s correlation coefficients, principal component analysis (PCA), biplot, MGIDI analysis, and diagrams were performed in R version 4.5.0 using metan, corrplot, factoextra, FactoMineR and ggplot2 packages, respectively.

## Results

In current study 230 wheat genotypes across six environments using grain yield under non-stress (Yp) and stress (Ys) conditions and three stress tolerance indices tolerance index (TOL), stress tolerance index (STI), and heat susceptibility index (HSI) were assessed. These parameters were used to characterize genotypic variation in yield performance and response to heat stress. The analyses included combined analysis of variance (ANOVA), correlation analysis, principal component analysis (PCA), and multi-trait genotype-ideotype distance index (MGIDI) analysis. The complete dataset of grain yield and stress tolerance indices for all genotypes across environments is provided in **Supplementary Table S2**.

Quantitative thermal characterization during grain filling was added across all three locations and both seasons (**Supplementary Table S3**). At Jabalpur, late sowing increased mean Tmax from 25.69 to 34.50 °C in 2021–22 and from 29.56 to 32.14 °C in 2022-23, with Tmax >30 °C occurring on 28 and 25 days, respectively. In Ludhiana, late sowing in 2021–22 resulted in the highest recorded thermal exposure (36.11 °C mean Tmax; 36 days >30 °C; 21 days >35 °C; 219.9 °C-days), whereas 2022–23 temperature records were available only weekly and are therefore reported as observation weeks. At PUSA, late sowing similarly increased thermal exposure, reaching 29 days >30 °C and 8 days >35 °C in 2021-22, and 22 days >30 °C in 2022-23. These results demonstrate clear differences in thermal exposure between normal- and late-sown conditions during grain filling.

### Combined analysis of variance for grain yield and stress tolerance indices

The combined analysis of variance (ANOVA) across six environments showed significant differences (*p*< 0.001) for Yp, Ys, TOL, and STI except HSI (**Table 2**). Significant environmental effects (*p*< 0.001) were observed for all traits and indices. The significant genotype effects for Yp and Ys indicate substantial genetic variation in grain yield under non-stress and heat-stress conditions, respectively. Significant variation for TOL and STI further indicates differences among genotypes in their responses to contrast environmental conditions, whereas the non-significant genotype effect for HSI suggests limited genotypic differentiation for these indices across the environments evaluated.

**Table 2:** Combined analysis of variance (ANOVA) for grain yield under non-stress (Yp) and stress (Ys) conditions and stress tolerance indices across environments.

	Mean square					
Source of variation	DF	Yp	Ys	TOL	STI	HSI
Genotype	229	4.87***	2.16***	1.32***	0.37***	1.26
Environment	5	126.18***	330.49***	120.30***	7.80***	6.05***
Residuals	1145	0.51	0.4	0.74	0.04	1.39

*, **, *** indicate significance at P< 0.05, P< 0.01, and P< 0.001, respectively; ns, not significant.

Environmental variation was pronounced across the six environments, with environment mean squares of 126.18 for Yp and 330.49 for Ys. The significant environmental effects indicate that growing conditions substantially influenced grain yield and the expression of stress-response traits. The larger environmental mean square for Ys than for Yp further indicates a stronger environmental influence on grain yield under stress conditions.

### Stress tolerance indices

In this study, based on yield under normal conditions and heat stress conditions, different stress indices like STI, TOL, and HSI were estimated (**Supplementary Table S2**). Stress tolerance indices revealed substantial variation among the 230 genotypes in their response to different sowing conditions. Genotypes G10037, G10030, G12015, G10018 and G10019 showed comparatively favorable combinations of stress yield and tolerance indices. Among these, G10037 showed a relatively high stress yield (Ys = 4.32) with low TOL (0.75) and HSI (0.34), together with an STI of 0.95, and exhibited only a 14.8% yield reduction under stress. Genotype G12015 also showed a low TOL (0.67), low HSI (0.42), and the low yield reduction among these high-ranking entries (13.5%), although its STI was moderate (0.89). Genotypes G10030 and G10018 combined relatively high stress yield (4.50 and 4.56, respectively) with STI values of 1.09 and 1.08 and low HSI values of 0.40 and 0.58. Similarly, G10019 maintained high stress yield (4.44) with STI = 1.04 and HSI = 0.45.

### Correlation between grain yield and stress tolerance indices

Pearson correlation analysis showed a strong positive association between grain yield under non-stress (Yp) and stress (Ys) conditions (*r* = 0.88, *P*< 0.001; **Figure 1****;** **Table 3** indicating that genotypes with greater yield potential generally maintained higher yield under stress. STI was very strongly correlated with both Yp (*r* = 0.96, *P*< 0.001) and Ys (*r* = 0.96, *P*< 0.001), consistent with its derivation from yield performance under both conditions. TOL showed positive associations with Yp (*r* = 0.80, *P*< 0.001) and Ys (*r* = 0.41, *P*< 0.001), whereas HSI was moderately correlated with Yp (*r* = 0.43, *P*< 0.001) but showed no significant association with Ys (*r* = 0.10, *P* = 0.143). TOL and HSI were strongly correlated (*r* = 0.71, *P*< 0.001), indicating substantial overlap in the susceptibility information captured by these indices. Overall, the strong correlations among Yp, Ys, and the derived indices highlight their mathematical dependence and indicate that the indices should not be interpreted as independent measures in subsequent multivariate analyses.

**Figure 1:**
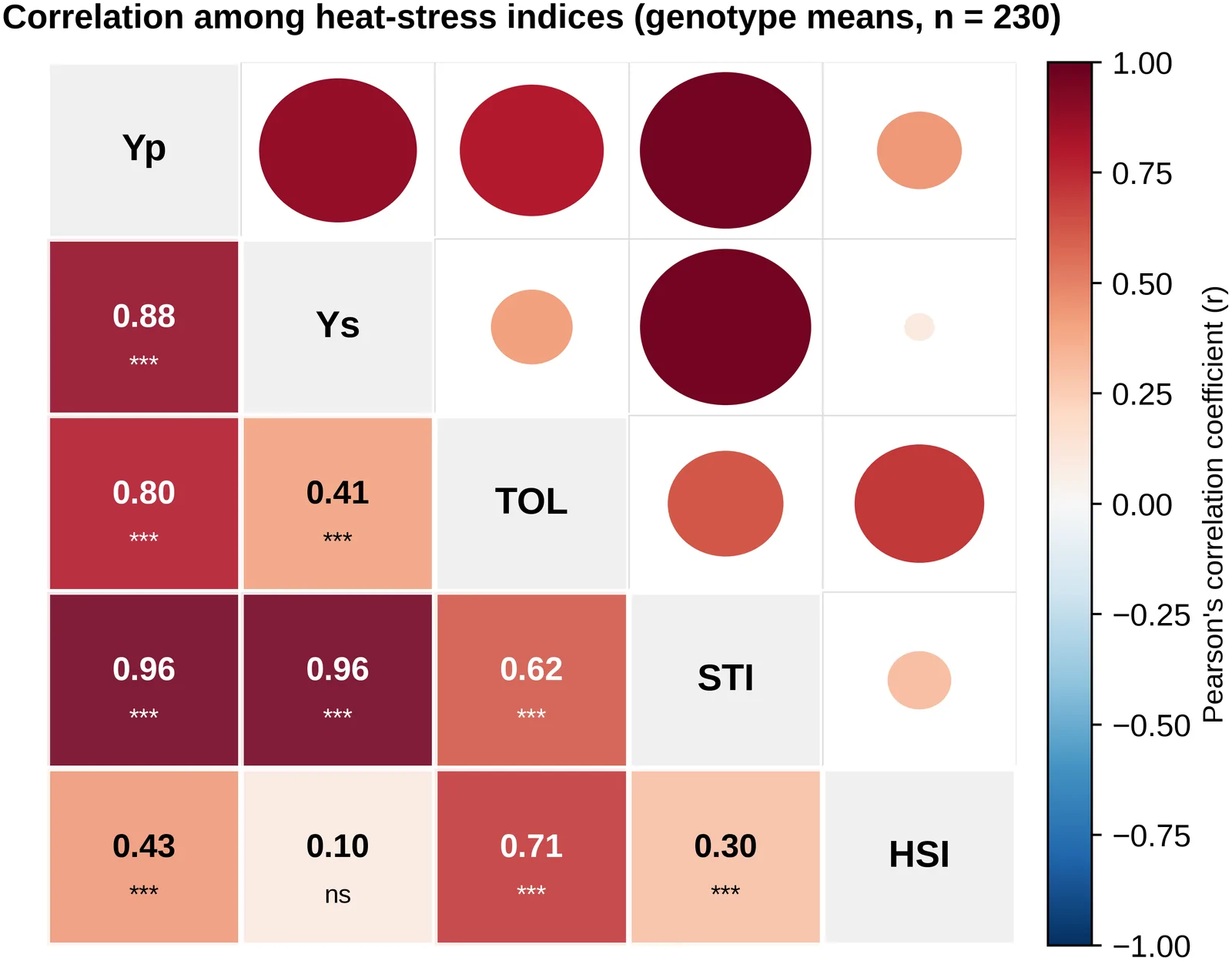
Pearson correlation matrix among grain yield and heat-stress tolerance indices across 230 wheat genotypes. Yp, grain yield under non-stress conditions; Ys, grain yield under heat-stress conditions; TOL, tolerance index; STI, stress tolerance index; HSI, heat susceptibility index. Significance levels are indicated by *** (*P*< 0.001) and ns (not significant).

**Table 3:** Pearson correlation coefficients among grain yield under non-stress (Yp) and heat-stress (Ys) conditions and derive stress tolerance indices (TOL, STI, and HSI) across 230 genotype means.

Trait	Yp	Ys	TOL	STI	HSI
Yp	1	0.880***	0.796***	0.964***	0.433***
Ys		1	0.413***	0.963***	0.097 ns
TOL			1	0.622***	0.708***
STI				1	0.302***
HSI					1

*, **, *** = p< 0.05, 0.01, 0.001; ns, not significant (df = 228).

### Principal component analysis

Principal component analysis (PCA) based on standardized TOL, STI, and HSI values showed that PC1 and PC2 explained 70.2% and 23.4% of the total variation, respectively, accounting for 93.6% cumulatively (**Figure 2**). PC1 was primarily associated with STI and TOL, whereas PC2 was dominated by HSI, indicating contrast dimensions of stress-response variation among genotypes. The biplot broadly separated the three clusters, with Cluster II positioned toward higher TOL and HSI values, whereas Cluster I was distributed mainly toward negative PC1 values. Cluster III occupied an intermediate to positive PC1 region and included G10018, G10037, and G12015, which also exhibited favorable stress-response in the clustering and multi-trait selection analyses. Similar separation of productivity- and susceptibility-related dimensions has been reported in PCA-based characterization of wheat genotypes under heat stress (Lamba et al., 2023; Kumar et al., 2024). PCA incorporating grain yield and stress tolerance indices further summarized the multivariate structure of the evaluated genotypes (**Tables 4**, **5**; **Figure 3**). The first two components explained 71.42% and 23.81% of the total variation, respectively, accounting for 95.23% cumulatively. PC1 showed strong positive loadings for Yp (0.992), STI (0.951), Ys (0.848), and TOL (0.821), defining a dominant productivity-related axis integrating yield performance under non-stress and heat-stress conditions. In contrast, HSI showed its strongest loading on PC2 (0.788), indicating that this component primarily represented susceptibility-related variation. This productivity–susceptibility structure is consistent with previous PCA-based analyses of wheat heat-stress tolerance indices (Lamba et al., 2023; Kumar et al., 2024; Gaikwad et al., 2025).

**Figure 2:**
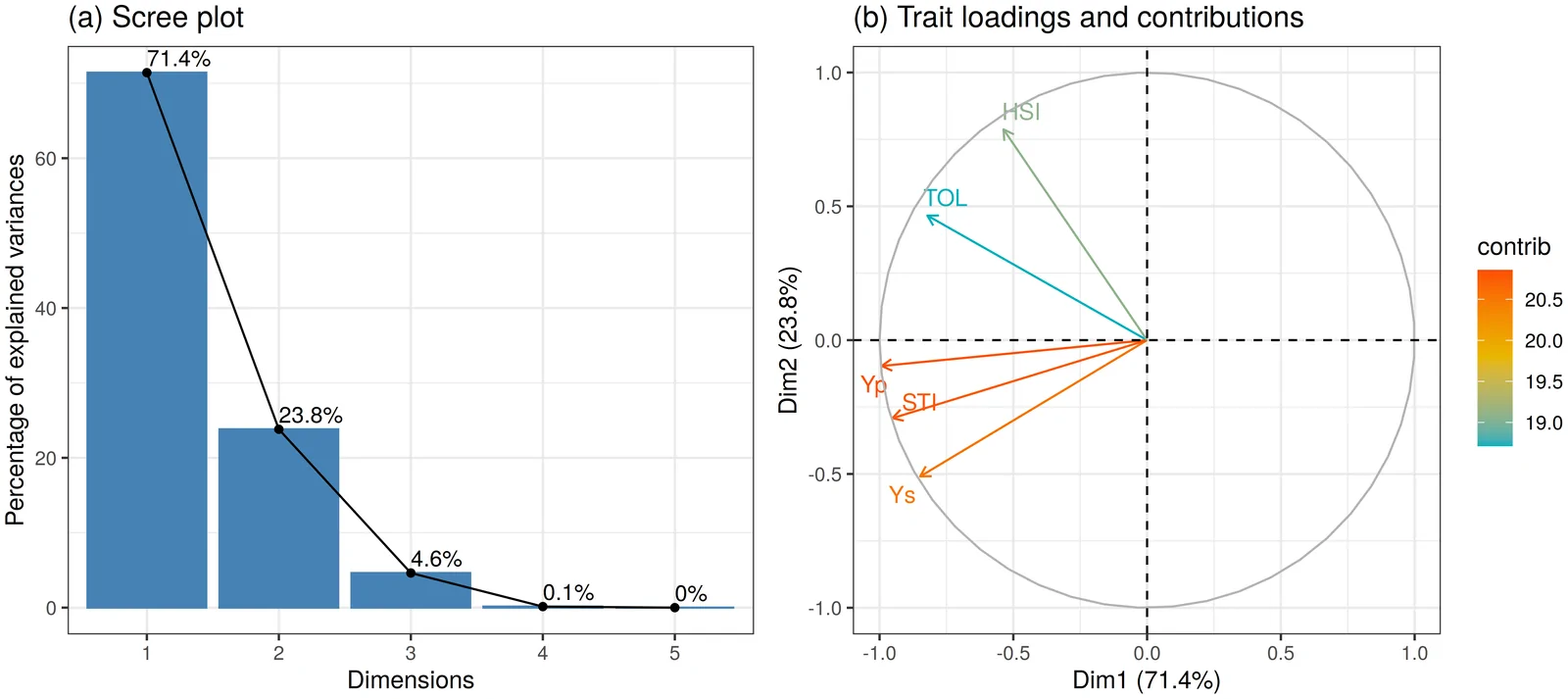
Principal component biplot of 230 wheat genotypes based on standardized TOL, STI, and HSI values, showing genotype distribution according to Ward’s hierarchical clusters. PC1 and PC2 explained 70.2% and 23.4% of the total variation, respectively. Arrows indicate the direction of the index variables, and shaded regions represent the three Ward clusters. Highlighted genotypes represent entries jointly identified within the top-30% selection threshold for STI, TOL, and HSI. The two standard checks are shown separately.

**Table 4:** Eigenvalues and percentage of variance explained by the principal components derived from Yp, Ys, TOL, STI, and HSI, Eigenvalue, variance (%) and cumulative variance (%) indicate the variation explained by each component.

PC	Eigenvalue	Variance_pct	Cumulative_pct
PC1	3.571	71.42	71.42
PC2	1.191	23.81	95.23
PC3	0.231	4.63	99.86
PC4	0.007	0.14	100
PC5	0	0	100

**Table 5:** Trait loadings of Yp, Ys, TOL, STI, and HSI on the principal components, Values represent standardized trait loadings; larger absolute values indicate greater contributions to the respective principal components.

Trait	PC1	PC2	PC3	PC4
Yp	0.992	-0.096	0.079	-0.027
Ys	0.848	-0.509	-0.14	-0.041
TOL	0.821	0.465	0.33	0
STI	0.951	-0.292	-0.071	0.068
HSI	0.537	0.788	-0.302	-0.004

**Figure 3:**
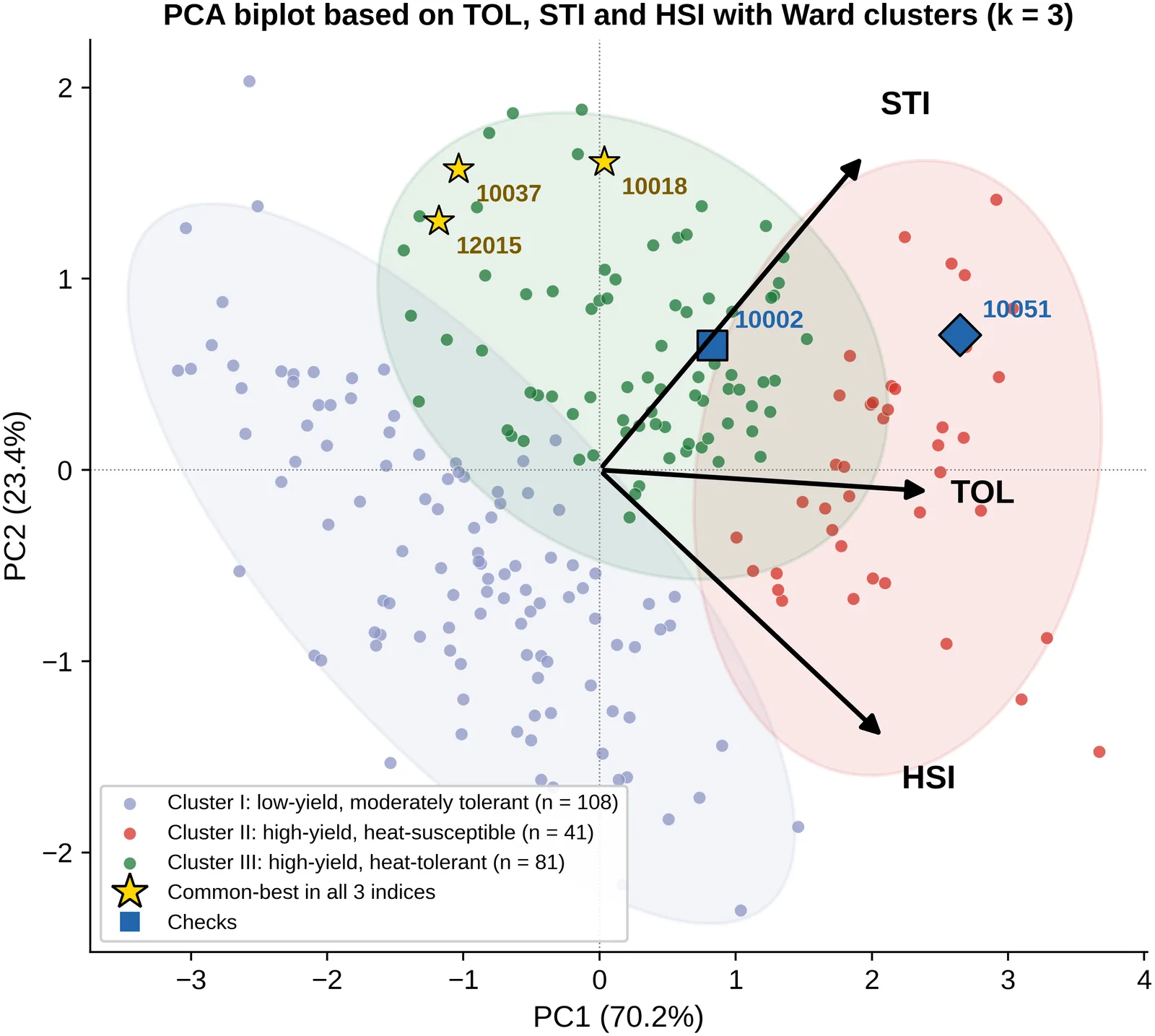
Principal component analysis of grain yield and heat-stress tolerance indices across 230 wheat genotypes, showing **(a)** the percentage of variance explained by each principal component and **(b)** trait loadings and contributions to the first two principal components. Yp, grain yield under non-stress conditions; Ys, grain yield under heat-stress conditions; TOL, tolerance index; STI, stress tolerance index; HSI, heat susceptibility index. PC1 and PC2 explained 71.4% and 23.8% of the total variation, respectively.

### Cluster analysis

Hierarchical clustering based on standardized TOL, STI, and HSI values separated the 230 wheat genotypes into three clusters using Euclidean distance and Ward’s method (**Figure 4**; **Table 6**). Silhouette analysis supported cluster solutions from *k* = 2 to 4, and *k* = 3 was selected based on interpretability. Cluster I comprised 108 genotypes and was characterized by the lowest mean Yp (3.981) and STI (0.487), with intermediate TOL (1.090) and HSI (0.767). Cluster II comprised 41 genotypes and showed the highest Yp (5.955), but also the highest TOL (2.093) and HSI (1.411), indicating high yield potential accompanied by greater stress susceptibility. Cluster III comprised 81 genotypes and exhibited a comparatively favorable combination of Yp (5.311), STI (0.878), TOL (1.387), and HSI (0.791), indicating a group with relatively favorable stress-response characteristics while maintaining moderate-to-high yield potential. Several genotypes within Cluster III showed particularly favorable combinations of the evaluated indices, including G10018, G10019, G10030, G10037, G10040, G10071, G12015, G12020, G12027, G12029, and G12031. The clustering therefore provided a population-level classification of genotypes according to their stress-response profiles and served as a basis for subsequent multi-trait selection using MGIDI.

**Figure 4:**
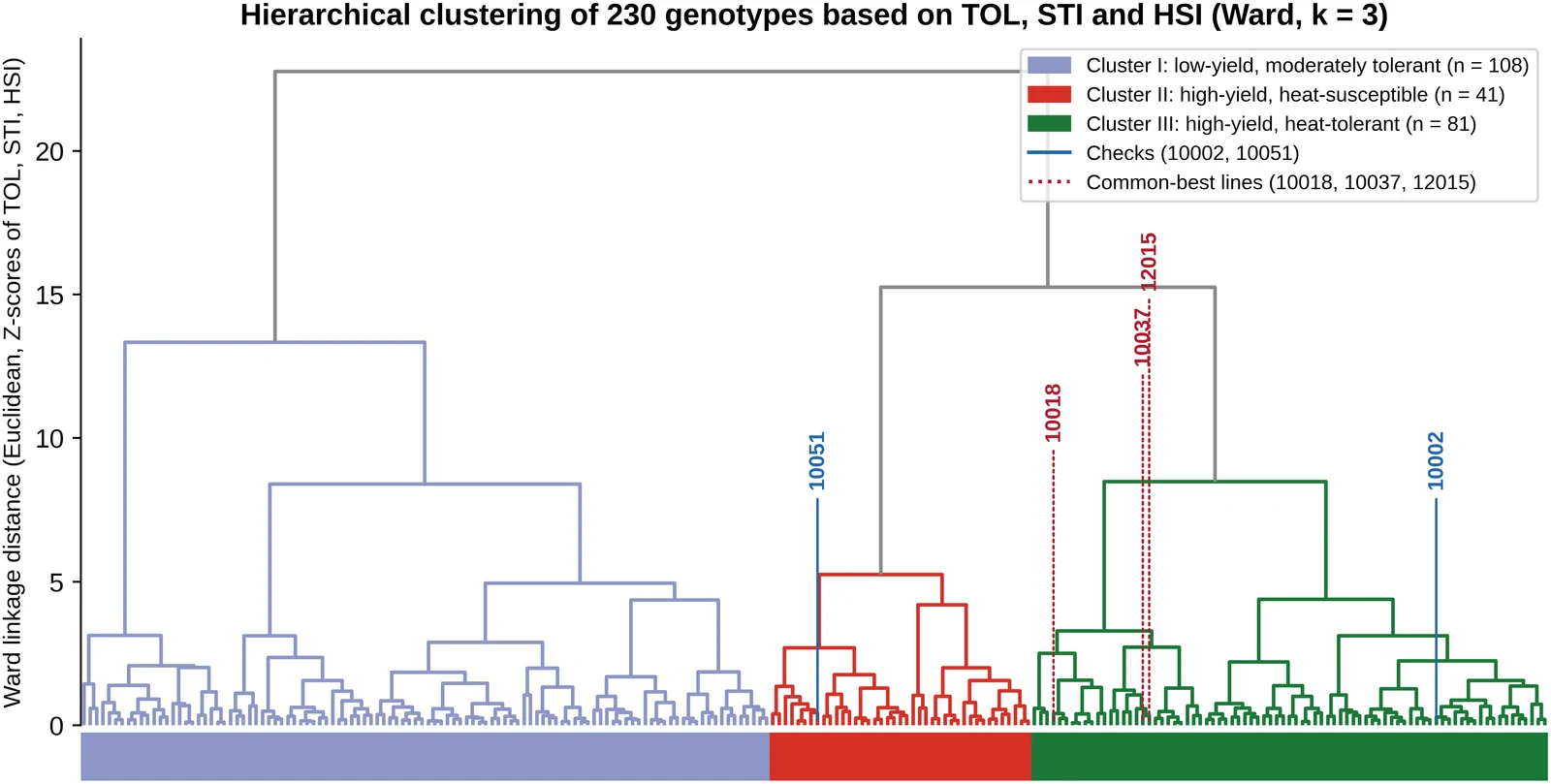
Hierarchical clustering of 230 wheat genotypes based on standardized TOL, STI, and HSI values using Ward’s method. Euclidean distance was calculated from Z-score-standardized TOL, STI, and HSI values. The three clusters were selected based on silhouette-supported solutions (k = 2-4) and interpretability. Checks 10002 and 10051 and the highlighted genotypes 10018, 10037, and 12015 are indicated separately.

**Table 6:** Cluster means for 230 wheat genotypes obtained by Ward’s hierarchical clustering based on TOL, STI, and HSI (k = 3).

Cluster	Description	n	TOL	STI	HSI	Yp	Ys
1	I: low-yield, moderately tolerant	108	1.09	0.487	0.767	3.981	2.891
2	II: high-yield, heat-susceptible	41	2.093	0.981	1.411	5.955	3.862
3	III: high-yield, heat-tolerant	81	1.387	0.878	0.791	5.311	3.924

Clustering was performed using Euclidean distance on Z-score-standardized TOL, STI, and HSI values with Ward’s method. Silhouette analysis supported k = 2-4; k = 3 was selected based on interpretability.

### *MGIDI-based* multi-trait selection

MGIDI analysis ranked all 230 genotypes according to their distance from the defined multi-trait ideotype, with lower MGIDI values indicating more desirable overall performance. At a 15% selection intensity, 34 genotypes were selected. The highest-ranked genotypes were G11019, G10037, G10030, G10071, G11066, G12015, G10019, G10018, G12027, and G12029, with MGIDI values ranging from (2.0646-2.6615) (**Figure 5**). Mainly most of Genotypes 28 of the 34 MGIDI-selected genotypes belonged to Cluster III, whereas six occurred in Cluster I and none were assigned to Cluster II. This substantial overlap between the clustering and MGIDI approaches indicates that the genotypes identified by multi-trait selection were predominantly concentrated within the cluster characterized by a comparatively favorable stress-response profile. Particularly consistent candidates included G10018, G10037, and G12015, which were highlighted in the clustering analysis and ranked among the leading MGIDI-selected genotypes. The two checks, G10002 and G10051, were not selected by MGIDI, ranking 60th and 127th, respectively (Hasan et al., 2024).

**Figure 5:**
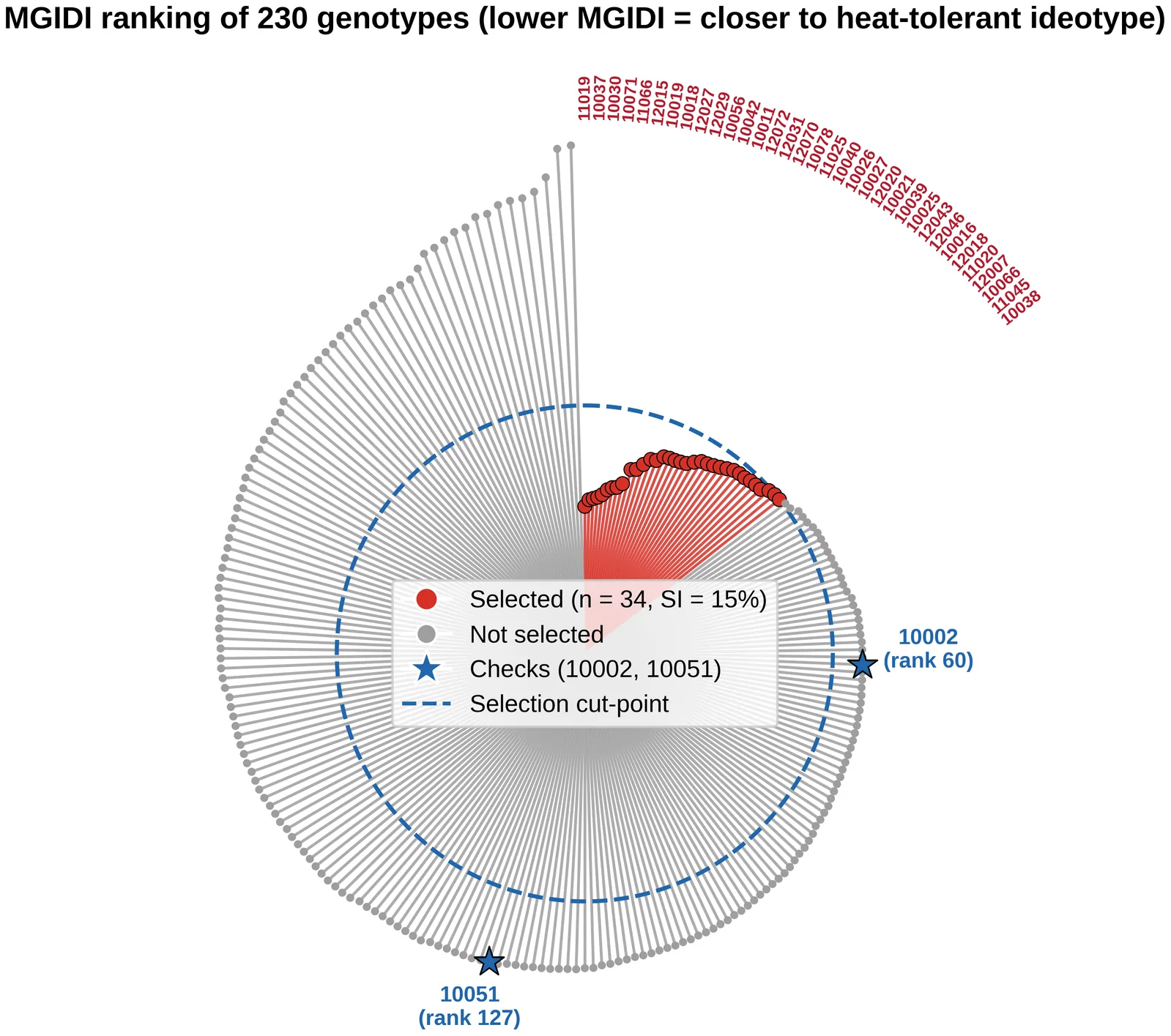
MGIDI ranking of ranking genotypes for multi-trait selection under heat-stress conditions. Genotypes are ranked in ascending order of MGIDI, with lower values indicating greater proximity to the specified multi-trait ideotype. Red and grey symbols indicate selected and non-selected genotypes, respectively; the dashed line represents the 15% selection cut-point (n = 34).

### Concordance among index-based selection approaches

Joint ranking of the 230 genotypes using STI (higher is desirable), TOL (lower is desirable), and HSI (lower is desirable) progressively narrowed the subset showing favorable profiles across all three indices. There is not any genotype met the top 15% criterion simultaneously for all three indices, whereas G10037 was the only genotype identified at the top 25% threshold. At the top 30% threshold, G10018, G10037, and G12015 were jointly identified, increasing to six genotypes at 35% and ten genotypes at 40% (**Table 7**). A complementary mean-rank analysis of STI, TOL, and HSI identified G10037, G11019, G12015, G10030, and G12027 as the five highest-ranked genotypes. Among the 20 highest-ranked genotypes, 16 were also selected by MGIDI, indicating substantial concordance between index-based ranking and multi-trait selection. G10037, G11019, G12015, G10030, G12027, G10018, and G10071 showed particularly consistent ranking patterns across the approaches (**Table 8**). Similar studies in wheat have used rank-based selection across multiple stress-tolerance indices to prioritize genotypes under contrasting stress conditions (Semahegn et al., 2020; Lamba et al., 2023), while integrated approaches combining clustering and MGIDI have further demonstrated the value of multivariate selection for identifying favorable genotype profiles (Hasan et al., 2024).

**Table 7:** Simultaneous selection of wheat genotypes based on STI, TOL, and HSI at different selection thresholds, where n indicates the number of genotypes satisfying the specified selection criterion simultaneously across all three indices.

Criterion	n	Genotypes
Top 15% of each index simultaneously	0	none —STI–TOL trade-off (r = 0.62)
Top 25% of each index simultaneously	1	10037
Top 30% of each index simultaneously	3	10018, 10037, 12015
Top 35% of each index simultaneously	6	10018, 10030, 10037, 10056, 10071, 12015
Top 40% of each index simultaneously	10	10018, 10019, 10030, 10037, 10056, 10071, 12015, 12027, 12029, 12072

STI, stress tolerance index; TOL, tolerance index; HSI, heat susceptibility index. Higher STI and lower TOL and HSI values were considered desirablees.

**Table 8:** Top 20 wheat genotypes ranked by mean rank across STI, TOL, and HSI.specified favorable fraction for all three indices simultaneously.

Entry	STI_ rank	TOL_ rank	HSI_ rank	Mean_ rank	TOL	STI	HSI	Ys	Cluster	MGIDI_ rank	MGIDI_ sel
G10037	44	21	30	31.7	0.751	0.949	0.34	4.317	3	2	TRUE
G11019	104	6	3	37.7	0.574	0.757	-0.391	3.85	1	1	TRUE
G12015	65	13	35	37.7	0.667	0.892	0.414	4.278	3	6	TRUE
G10030	13	76	33	40.7	1.143	1.09	0.395	4.501	3	3	TRUE
G12027	81	23	23	42.3	0.775	0.837	0.269	4.086	3	9	TRUE
G10018	17	67	51	45	1.116	1.082	0.575	4.562	3	8	TRUE
G10071	49	74	13	45.3	1.13	0.928	0.129	4.226	3	4	TRUE
G10019	26	81	38	48.3	1.156	1.038	0.445	4.437	3	7	TRUE
G10056	71	28	50	49.7	0.798	0.877	0.572	4.2	3	11	TRUE
G12070	103	12	44	53	0.648	0.764	0.486	3.844	3	16	TRUE
G10042	107	38	20	55	0.894	0.748	0.191	3.817	3	12	TRUE
G12072	46	39	81	55.3	0.899	0.939	0.757	4.236	3	14	TRUE
G11066	50	117	10	59	1.345	0.925	0.032	4.184	3	5	TRUE
G11033	132	15	31	59.3	0.689	0.603	0.363	3.39	1	36	FALSE
G12018	167	1	12	60	0.341	0.512	0.069	3.326	1	29	TRUE
G11025	144	43	2	63	0.926	0.555	-0.395	3.25	1	18	TRUE
G10073	163	9	18	63.3	0.615	0.52	0.163	3.205	1	37	FALSE
G10076	152	11	28	63.7	0.634	0.54	0.331	3.279	1	41	FALSE
G12029	87	86	19	64	1.193	0.821	0.167	3.955	3	10	TRUE
G12062	178	2	17	65.7	0.463	0.484	0.158	3.17	1	40	FALSE

Green = also MGIDI-selected (16/20 agree — strong cross-validation of the MGIDI result).

Higher STI and lower TOL and HSI values were considered desirable, Rankings were assigned according to the desirability direction of each index, with higher STI and lower TOL and HSI considered favorable. Mean rank represents the average of the individual STI, TOL, and HSI ranks. Green shading indicates genotypes that were also selected by MGIDI at 15% selection intensity.

## Discussion

Terminal heat stress is a major constraint to wheat productivity, particularly when elevated temperatures coincide with reproductive development and grain filling. In the present study, significant genotypic variation for grain yield under both non-stress (Yp) and heat-stress (Ys) conditions demonstrated substantial diversity in yield response within the evaluated panel. The stronger environmental effect observed for Ys than for Yp further indicates that grain yield under terminal heat stress was more sensitive to variation in growing conditions, highlighting the importance of multi-environment evaluation for identifying genotypes with favorable stress-response profiles. There were some genotypes and environmental effects have been reported in wheat evaluated under contrasting terminal heat conditions (Lamba et al., 2023; Poudel et al., 2021).

The relationships between Yp, Ys, and the derived indices require careful interpretation because TOL, STI, and HSI are mathematical transformations of Yp and Ys rather than independent measurements. Accordingly, the strong correlations observed among these variables primarily reflect shared information and do not necessarily indicate independent evidence of index validity. STI showed strong associations with both Yp and Ys, consistent with its expression as a productivity-oriented index incorporating performance under both conditions. TOL and HSI were also strongly associated, reflecting their shared emphasis on yield reduction under stress. In contrast, the absence of a significant association between HSI and Ys indicates that relative susceptibility is not equivalent to absolute yield under stress. These relationships emphasize that the indices should be interpreted according to their distinct selection objectives rather than treated as independent measures of heat tolerance.

The contrasting discriminatory ability of the indices further supports selective more appropriately viewed as indiscriminate use of derived measures. Significant genotypic variation was detected for TOL and STI, whereas HSI did not significantly differentiate genotypes in the present population. Thus, HSI may provide information on relative susceptibility but had limited discriminatory value under the conditions evaluated. STI, by incorporating yield information from both non-stress and stress conditions, provided a useful productivity-oriented criterion for prioritization. These indices have also been taken as a selection criterion of heat tolerance lines in wheat (Poudel et al., 2021). Nevertheless, its strong dependence on Yp and Ys means that its association with these variables is not sufficient to establish evidence that STI captures an independent biological component of heat tolerance. Previous wheat studies have likewise shown that the relative usefulness of stress indices can vary with population characteristics and stress intensity (Poudel et al., 2021; Lamba et al., 2023). Collectively, these findings support the targeted use of complementary indices according to their selection objectives.

PCA further summarized the statistical structure of the yield-derived variables. The first two components accounted for 95.23% of the total variance, with Yp, Ys, STI, and TOL contributing strongly to the principal productivity-related dimension, whereas HSI contributed predominantly to the second, susceptibility-related dimension (**Table 4**). This structure is consistent with the tendency of stress-index datasets to separate productivity and stress-response characteristics in multivariate analyses (Lamba et al., 2023; Gaikwad et al., 2025). Despite this the high cumulative variance cannot by itself demonstrate evidence of independent biological dimensions, because the indices are mathematically derived from the same two yield variables. PCA therefore served primarily to summarize and visualize relationships among the yield-response measures instead of to validate the indices or establish independent dimensions of heat tolerance.

Hierarchical clustering provided a complementary population-level classification of the genotypes according to their standardized TOL, STI, and HSI profiles. The three clusters represented contrast combinations of yield potential and stress-response characteristics, with Cluster III showing a comparatively favorable combination of yield potential, STI, TOL, and HSI, whereas Cluster II combined high yield potential with less favorable stress-response characteristics. This classification illustrates the trade-off that can arise when productivity and stress-response attributes are considered simultaneously and provides a useful framework for narrowing the germplasm pool before genotype-level selection. A key point is that clustering should be interpreted as a classification of patterns within the same yield-derived information, instead of as independent confirmation of heat tolerance.

Joint ranking provided an additional basis for identifying genotypes that simultaneously satisfied the desirability criteria of STI, TOL, and HSI. The absence of a genotype meeting the top-15% threshold for all three indices and the progressive increase in common genotypes at less stringent thresholds indicate that simultaneous selection can substantially narrow the candidate set when multiple, partially contrasting index objectives are imposed. G10037 was the only genotype identified at the top-25% threshold, while G10018, G10037, and G12015 were jointly identified at the top-30% threshold. Their recurrence suggests favorable positioning across the different index criteria, although this concordance remains based on overlapping yield-derived information instead of independent measurements (Olivoto and Nardino, 2021).

MGIDI provided a further genotype-level prioritization according to the specified multi-trait ideotype. At a 15% selection intensity, 34 genotypes were selected, and 28 of these belonged to Cluster III, whereas none occurred in the high-yield but less favorable stress-response cluster. This overlap indicates that MGIDI-selected genotypes were predominantly concentrated within the cluster characterized by a comparatively favorable stress-response profile. Despite this, the agreement does not provide evidence of independent validation because both clustering and MGIDI incorporate overlapping information from the same yield-derived indices. Rather, the two approaches provide complementary perspectives: clustering describes population-level structure, whereas MGIDI ranks individual genotypes according to their overall proximity to the specified selection target.

The repeated identification of G10037, G10018, and G12015 across joint index ranking, clustering, and MGIDI provides a practical basis for prioritizing these entries for further evaluation. Their recurrence across analytical perspectives indicates that their favorable profiles were not restricted to a single selection criterion, while the substantial overlap between the top mean-ranked genotypes and MGIDI-selected entries further supports consistency in genotype prioritization. Despite these findings such concordance does not necessarily indicate proof of stable genetic heat tolerance because the analytical approaches rely largely on the same yield-derived information. These genotypes are therefore best regarded as promising candidates with favorable stress-response profiles under the tested environments, as opposed to confirmed heat-tolerant donor genotypes. From a breeding perspective, the results demonstrate the value of integrating complementary statistical summaries to reduce a large germplasm panel to a manageable set of candidates while retaining explicit selection criteria for productivity and stress response. At the same time, the yield-based nature of the present analysis limits inference about the physiological mechanisms underlying heat resilience. Confirmation of the stability and genetic value of the prioritized genotypes will require independent multi-environment testing, preferably incorporating physiological, phenological, and, where appropriate, genomic information. Such integration would help determine whether the favorable yield responses identified here are consistently expressed across environments and are associated with specific mechanisms of terminal heat adaptation.

## Conclusion

In this study, substantial genetic variability was noted for Yp, Ys, TOL and STI, in contrast HSI had not significant at the selection level. TOL and STI could be used for the selection of heat tolerant lines among the studied bread wheat genotypes grown in six environments characterized by different conditions. The results of this study also emphasize one more issue of methodological importance. Stress indices obtained from Yp and Ys are dependent measures and thus correlations between them cannot be viewed only in terms of their magnitude. The same concerns the principal components analysis performed. For this reason, the selection potential of stress indices based on Yp and Ys was assessed mainly from the point of view of the complementarity of the methods and consistency of the ranking results. An encouraging result is the consistency of the methods. The same genotypes were repeatedly detected by several indices (G10037, G10018 and G12015), and 28 of 34 genotypes selected by the MGIDI procedure at 15% selection intensity belong to a less stressful response cluster. These genotypes can be considered as potential candidates for further investigation and breeding program in the terminal heat-stress environment.

## Data Availability

The datasets presented in this study can be found in online repositories. The names of the repository/repositories and accession number(s) can be found in the article/**Supplementary Material**.

## Supplementary material

Supplementary Table 1 List of 230 bread wheat genotypes (accession codes) used in the present study, check cultivars are highlighted.

Supplementary Table 2 Computation of heat tolerance indices; Grain yield/plot (g), Yp and Ys under normal and stress conditions and stress tolerance indices of different wheat genotypes. STI stress tolerance index, Yp grain yield of genotypes under normal condition, TOL tolerance index, HSI Heat Susceptibility Index.

Supplementary Table 3 Quantitative thermal characterization during grain filling added across all three locations and both seasons.

Supplementary Figure 1 Comprehensive comparison of weekly maximum (upper) and minimum (lower) temperatures (°C) at Ludhiana (LDH), Jabalpur (JBL), and Pusa (PUSA) across two consecutive crop cycles (2021–22 and 2022-23). Highlighting variation in temperature patterns and terminal heat-stress intensity across locations.
