# Supplementary material for: Integrating heat stress tolerance indices based on grain yield for the selection of resilient wheat genotypes
Source: Front Plant Sci. 2026 Oct 27;17:1949670. doi: 10.3389/fpls.2026.1949670 (PMC13748455; doi:10.3389/fpls.2026.1949670)
Supplement: Supplementary file 1 [file Supplementaryfile1.docx]

Supplementary Figure 1. Comprehensive comparison of weekly maximum (upper) and minimum (lower) temperatures (°C) at Ludhiana (LDH), Jabalpur (JBL), and Pusa (PUSA) across two consecutive crop cycles (2021-22 and 2022-23). Highlighting variation in temperature patterns and terminal heat-stress intensity across locations.
