# Supplementary material for: Integrating heat stress tolerance indices based on grain yield for the selection of resilient wheat genotypes
Source: Front Plant Sci. 2026 Oct 27;17:1949670. doi: 10.3389/fpls.2026.1949670 (PMC13748455; doi:10.3389/fpls.2026.1949670)
Supplement: Supplementary file 2 [file Table1.docx]

**Supplementary Table 1:** List of 230 bread wheat genotypes (accession codes) used in the present study, check cultivars are highlighted.

| **Entry** | **NBPGR Accession** | **Entry** | **NBPGR Accession** | **Entry** | **NBPGR Accession** |
| --- | --- | --- | --- | --- | --- |
| 10001 | 8 | 11001 | 466 | 12001 | 652 |
| 10002 | RAJ3765 (Check) | 11002 | RAJ3765 (Check) | 12002 | RAJ3765 (Check) |
| 10003 | 9 | 11003 | 468 | 12003 | 653 |
| 10004 | 11 | 11004 | 479 | 12004 | 654 |
| 10005 | 16 | 11005 | 484 | 12005 | 682 |
| 10006 | 20 | 11006 | 485 | 12006 | 697 |
| 10007 | 21 | 11007 | 488 | 12007 | 702 |
| 10008 | 22 | 11008 | 491 | 12008 | 712 |
| 10009 | 26 | 11009 | 497 | 12009 | 713 |
| 10010 | 28 | 11010 | 499 | 12010 | 714 |
| 10011 | DBW107 (Check) | 11011 | DBW107 (Check) | 12011 | DBW107 (Check) |
| 10012 | 30 | 11012 | 501 | 12012 | 715 |
| 10013 | 31 | 11013 | 502 | 12013 | 748 |
| 10014 | 32 | 11014 | 503 | 12014 | 749 |
| 10015 | 33 | 11015 | 504 | 12015 | 752 |
| 10016 | 37 | 11016 | 506 | 12016 | 753 |
| 10017 | 40 | 11017 | 507 | 12017 | 754 |
| 10018 | 51 | 11018 | 513 | 12018 | 756 |
| 10019 | 52 | 11019 | 515 | 12019 | 763 |
| 10020 | 53 | 11020 | 517 | 12020 | 799 |
| 10021 | 54 | 11021 | 520 | 12021 | 812 |
| 10022 | 55 | 11022 | 523 | 12022 | 815 |
| 10023 | 56 | 11023 | 532 | 12023 | 818 |
| 10024 | 58 | 11024 | 533 | 12024 | 819 |
| 10025 | 59 | 11025 | 534 | 12025 | 821 |
| 10026 | 60 | 11026 | 535 | 12026 | 825 |
| 10027 | 61 | 11027 | 536 | 12027 | 844 |
| 10028 | 62 | 11028 | 538 | 12028 | 846 |
| 10029 | 63 | 11029 | 541 | 12029 | 868 |
| 10030 | DBW71 (Check) | 11030 | DBW71 (Check) | 12030 | DBW71 (Check) |
| 10031 | 80 | 11031 | 5130 | 12031 | 869 |
| 10032 | 82 | 11032 | 545 | 12032 | 870 |
| 10033 | 83 | 11033 | 546 | 12033 | 873 |
| 10034 | 84 | 11034 | 547 | 12034 | 875 |
| 10035 | 99 | 11035 | 549 | 12035 | 880 |
| 10036 | 100 | 11036 | 550 | 12036 | 883 |
| 10037 | 102 | 11037 | 551 | 12037 | 884 |
| 10038 | 103 | 11038 | 552 | 12038 | 902 |
| 10039 | 106 | 11039 | 553 | 12039 | 906 |
| 10040 | 110 | 11040 | 556 | 12040 | 910 |
| 10041 | 111 | 11041 | 557 | 12041 | 930 |
| 10042 | 117 | 11042 | 560 | 12042 | 931 |
| 10043 | 160 | 11043 | 564 | 12043 | 934 |
| 10044 | 161 | 11044 | 565 | 12044 | 936 |
| 10045 | 162 | 11045 | 566 | 12045 | 937 |
| 10046 | 165 | 11046 | 572 | 12046 | 938 |
| 10047 | 197 | 11047 | 573 | 12047 | 956 |
| 10048 | 198 | 11048 | 575 | 12048 | 978 |
| 10049 | 199 | 11049 | 576 | 12049 | 979 |
| 10050 | 210 | 11050 | 578 | 12050 | 991 |
| 10051 | HD2932 (Check) | 11051 | HD2932 (Check) | 12051 | HD2932 (Check) |
| 10052 | 214 | 11052 | 579 | 12052 | 992 |
| 10053 | 215 | 11053 | 581 | 12053 | 993 |
| 10054 | 216 | 11054 | 584 | 12054 | 998 |
| 10055 | 219 | 11055 | 585 | 12055 | 1022 |
| 10056 | 221 | 11056 | 588 | 12056 | 1023 |
| 10057 | 225 | 11057 | 590 | 12057 | 1026 |
| 10058 | 235 | 11058 | 591 | 12058 | 1027 |
| 10059 | 237 | 11059 | 592 | 12059 | 1031 |
| 10060 | 263 | 11060 | 595 | 12060 | 1040 |
| 10061 | 287 | 11061 | 596 | 12061 | 1047 |
| 10062 | 291 | 11062 | 597 | 12062 | 1062 |
| 10063 | 294 | 11063 | 598 | 12063 | 1358 |
| 10064 | 300 | 11064 | 600 | 12064 | 1113 |
| 10065 | 398 | 11065 | 601 | 12065 | 1117 |
| 10066 | 399 | 11066 | 602 | 12066 | 1119 |
| 10067 | 400 | 11067 | 603 | 12067 | 1159 |
| 10068 | 401 | 11068 | 604 | 12068 | 1166 |
| 10069 | 429 | 11069 | 606 | 12069 | 1171 |
| 10070 | 887 | 11070 | 615 | 12070 | 1178 |
| 10071 | 432 | 11071 | 616 | 12071 | 1180 |
| 10072 | 441 | 11072 | 625 | 12072 | 1183 |
| 10073 | 442 | 11073 | 632 | 12073 | 1184 |
| 10074 | PBW771 (Check) | 11074 | PBW771 (Check) | 12074 | PBW771 (Check) |
| 10075 | 443 | 11075 | 633 | 12075 | 1185 |
| 10076 | 445 | 11076 | 643 | 12076 | 1187 |
| 10077 | 446 | 11077 | 644 | 12077 | 1188 |
| 10078 | 447 | 11078 | 645 | 12078 | 1189 |
| 10079 | 448 | 11079 | 646 | 12079 | 1249 |
| 10080 | 453 | 11080 | 651 | 12080 | 1253 |

***Check cultivars: RAJ3765, HD2932. Pedigree information to be added before final submission.***
